# Treatment by a nurse practitioner in primary care improves the severity and impact of urinary incontinence in women. An observational study

**DOI:** 10.1186/s12894-015-0047-0

**Published:** 2015-06-12

**Authors:** Doreth T.A.M Teunissen, Marjolein M. Stegeman, Hans H. Bor, Toine A.L.M Lagro-Janssen

**Affiliations:** Department Primary and Community Care, Gender & Women’s Health, Radboud University Medical Centre Nijmegen, Internal postal code 118, P.O. Box 9101, , 6500 HB Nijmegen, The Netherlands

**Keywords:** Urinary Incontinence, Nurse Practitioner, Primary care, Quality of life

## Abstract

**Background:**

Urinary Incontinence (UI) is a common problem in women. The management of UI in primary care is time consuming and suboptimal. Shift of incontinence-care from General Practitioners (GP’s) to a nurse practitioner maybe improves the quality of care. The purpose of this observational (pre/post) study is to determine the effectiveness of introducing a nurse practitioner in UI care and to explore women’s reasons for not completing treatment.

**Methods:**

Sixteen trained nurse practitioners treated female patients with UI. All patients were examined and referred by the GP to the nurse practitioner working in the same practice. At baseline the severity of the UI (Sandvik-score), the impact on the quality of life (IIQ) and the impressed severity (PGIS) was measured and repeated after three months Differences were tested by the paired t and the NcNemar test.

Reasons for not completing treatment were documented by the nurse practitioner and differences between the group that completed treatment and the drop-out group were tested.

**Results:**

We included 103 women, mean age 55 years (SD 12.6). The Sandvik severity categories improved significantly *(P < 0.001),* as did the impact on daily life (2.54 points, *P = 0.012)*. Among the IIQ score the impact on daily activities increased 0.73 points *(P = 0.032)*, on social functioning 0.60 points *(P = 0.030)* and on emotional well-being 0.63 points *(P = 0.031)*. The PGIS-score improved in 41.3 % of the patients.

The most important reasons for not completing the treatment were lack of improvement of the UI and difficulties in performing the exercises. Women who withdraw from guidance by the nurse practitioner perceived more impact on daily life *(P = 0.036),* in particular on the scores for social functioning *(P = 0.015)* and emotional well-being *(P = 0.015)*.

**Conclusion:**

Treatment by a trained nurse practitioner seems positively affects the severity of the UI and the impact on the quality of life. Women who did not complete treatment suffer from more impact on quality of life, experience not enough improvement and mention difficulties in performing exercises.

## Background

Urinary Incontinence (UI) is a common problem in women with a prevalence varying from 25 % to 50 % [1, 2]. The impact of UI on daily life differs amongst patients. The highest impact is found on emotional wellbeing and public activities [3]. In addition, in the Netherlands annually €155 million is spent just on incontinence pads [4].

According to the guideline ‘Urinary incontinence’ of the The Dutch College of General Practitioners the prevalent treatment for stress- and urge-incontinence is pelvic floor exercises and bladder training respectively [5]. In mixed incontinence the treatment starts with the type of incontinence that is experienced by the patient as most disabling.

Adequate UI management according to the guideline is time-consuming. General practitioners (GP’s) may experience a lack of time and knowledge to provide adequate care [6]. Another obstacle is the low motivation of patients to perform the above mentioned interventions [7]. The most important factor for a successful treatment however is adherence to these interventions [8, 9]. Known factors for non-adherence to the exercises are difficulties in finding time to exercise and to recall the exercises [10]. An additional impediment is that some authors mention less motivation in the elderly patients to seek help and to accept the care required [7]. Therefore we need more insight into the reasons of patients in not accepting or completing treatment. This offers possibilities to improve the management of UI in primary care.

The last few decades there is a shift going on in care from GP’s to nurse practitioners to meet the rising demands for primary care. It appears that the nurse practitioners can produce the same quality of care and achieve comparable health outcomes as GP’s [11]. At the same time the care by nurse practitioners is patient-friendly and efficiently organized [12]. It was also shown to be cost-effective in general [13].

In a review about the role of a nurse in the care of patients with incontinence, nine of the twelve included studies reported a significant greater reduction of incontinence episode in the intervention group and patients were very satisfied. The nurses in the studies came from different countries and differed from a nurse with a bachelor of science in nursing degree to a nurse practitioner and the education differed from a full 6 weeks course till a short training by a local physiotherapist or behavioral psychologist [14, 15].

Recently also a Dutch study is conducted with nurse involved in the treatment of patients with UI [16]. Local Nurses working in de community and visiting patients at home, were shortly trained in the care of incontinence. They took tasks from the GP related to diagnostics, intervention and monitoring of patients with UI. The GP kept final responsibility. This study showed that involving a local nurse in UI primary care did reduce severity and impact of UI after three months but this improvement did not stay after twelve months. One of the problems mentioned in this study was the cooperation between the local nurse and GPs [12]. It was difficult to reach GPs to discuss patients and the referral rate from GP to the local nurse was low. Because of this we choose in our study to train nurse practitioners to guide patients with UI. A nurse practitioner is also a nurse but with additional a nurse practitioner diploma (after following a one year during education program focused on chronic care in the general practice). In the Netherlands the nurse practitioner is especially involved in the treatment and guidance of patients with chronic diseases such as Diabetes Mellitus en Chronic Pulmonary Disease with the core businesses to assess risk factors, to give health education and to perform motivational interviewing. A major advantage of the Dutch nurse practitioners is that they are part of the primary care team and they are working in the GP’s office. Therefore they are familiar to cooperate with the GP. This facilitates nurse practitioners to consult a GP when necessary, lowers thresholds for patients to seek help and possibly increases patient’s motivation. Another advantage of nurse practitioners is that they are involved in the treatment and guidance of patients with chronic diseases such as diabetes mellitus en chronic pulmonary diseases [17]. They can actively ask the patient with diabetes or chronic pulmonary disease about UI. Both morbidities are known with a high prevalence of UI.

Nevertheless the effectiveness of nurse practitioner in primary care trained in UI is not clear. Because of this we firstly set up a training program for nurse practitioners to enable them to take care of patients with UI. Secondly we conducted a clinical observational study to establish the effectiveness of both incontinence severity and quality of life. We also study characteristics and reasons of patients not completing the treatment program. Consequently, we formulated the following research questions:

- does the introduction of a nurse practitioner in the treatment of UI in women in primary care lead to an improvement of the UI severity and the quality of life after three months therapy?
- what are reasons for women not to complete the treatment program given by a trained nurse practitioner and are their differences in patient characteristics between the group that completed treatment and the drop-out group?

## Methods

### Design

We preformed an observational study.

### Setting

In this study a total of 16 nurse practitioners, already working with the GP in GP’s offices, in the eastern part of the Netherlands, undertook a training program in which they learned how to manage female patients with UI. The training program included 1.5 days course, several home assignments and refresher courses after three weeks, three and six months. The nurse practitioners were trained in tasks related to diagnostics, intervention and monitoring of incontinence based on the guideline ‘urinary incontinence’ of the The Dutch College of General Practitioners [5]. All nurse practitioners proved their competence after the course in an assessment. After the 1,5 days course the participants started to guide patients with UI within the beginning feedback from the GP and the members of the trainings board till they have proven their competencies in an individual assessment. At the refresher courses the nurse practitioners could discuss cases with experts and share their experiences. The training protocol is available upon request. The GP’s of the practices the nurse practitioners are working got a 3 h during education course to refresh the guideline and to explain with expertise they could expect from the nurse practitioners after the course.

### Participants

Women who asked for help in primary care for symptoms of stress-, urge- or mixed UI were included between June 2009 and December 2010. Patients who underwent an incontinence operation in de past, patients with a pelvic organ prolapse grade IV (Baden Walker), a gynecologic malignancy, a neurological disease or unstable mental disorder were excluded from the study.

### Data collection

The GP diagnosed prior to the referral to the nurse practitioner, the type of incontinence and excluded other causes of urinary incontinence like urinary tract infections or malignancies. After explanation about the study and informed consent of the patient to participate in the study the GP gave the patients information about the type of incontinence but did not explain treatment option. Because co-morbidities and use of medication can influence UI we also registered them. We compared these data with the prevalence of the same co-morbidities in a standard primary care population according to the Continuous Morbidity Registration Nijmegen (CMR) to known if our study population is a presentation of a general population. The CMR is a very reliable registration system which determines epidemiological numbers according to the incidence and prevalence of diseases in primary care for scientific research and education [18]. Because the CMR does not register medication for the aim of research we compared the use of diuretics and antidepressants with the corresponding diseases such as hypertension/heart failure and depression as registered in the CMR.

**Fig. 1:**
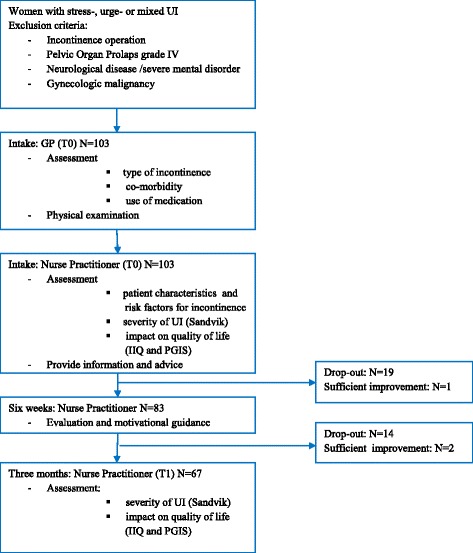
Flow chart study population

A view days after the consultation with the GP the patients had an appointment with the nurse practitioner. Prior to the first meeting with the nurse practitioner (T0), patients filled out questionnaires to assess the severity of the UI (Sandvik-score and the Patient Global Impression of Severity (PGIS) [19, 20] and its impact on the quality of life (Incontinence Impact Questionnaire) [21]. Patients’ variables like age and variables that may impact UI negatively like mobility constraints, micturition habits (taking time, have a good position on the toilet, do not postpone, voiding frequency 5 to 6 times a day), caffeine and alcohol intake were also mapped. Patients received information and advice and started with conservative treatment according to the Dutch guideline ‘urinary incontinence’ [5]. After six weeks, the patients returned to the nurse practitioner to evaluate the effect until then. The patients were motivated by the nurse to continue practicing and to follow the given advice. After three months (T1), patients filled out the same questionnaires as they did prior to the first meeting to evaluate the effect of the treatment (Fig. 1). We chose for a follow up of three months because the aim of the study is to study if the intervention seems to be effective and to study the feasibility of the intervention. The dutch guideline ‘Urinary incontinence’ of the The Dutch College of General Practitioners advise to evaluate the effectiveness of the therapy after three months. If the incontinence isn’t improved enough a referral to a physiotherapist of urologist/gynecologist can be considered. If the patient is satisfied with the effectiveness of the therapy it is important to explain that continuing exercises is needed to sustain this effect.

### Outcome measures

#### Primary outcome

The primary outcome measure was the severity of involuntary urine loss. . The severity of involuntary loss of urine was measured by the total Sandvik-score (i.e. quantity of urine loss multiplied by the frequency) [19]. The Sandvik-score of frequency are: never = 0, < 1 time a month = 1, a few times a month = 2, a few times a week = 3 and every day/night = 4. The Sandvik-score of quantity are: none = 0, drops = 1, little amount =2 and more = 3. The total Sandvik-score can be divided into five categories of severity; none = total Sandvik score 0, mild = total score 1–2, moderate = total score 3–6, severe = total score 8–9 and very severe = total score 12.

#### Secondary outcome

The secondary outcome measures were the impact of quality of life and patients appraisal of the severity of incontinence.

The impact on the quality of life was measured by the Incontinence Impact Questionnaire (IIQ), a questionnaire highly recommended by the International Consultation on Incontinence (= ICI) and is rated as Grade A [21] The IIQ measures the impact of the UI on daily activities (6 questions), social functioning (10 questions), emotional wellbeing (8 questions) and travelling/mobility (6 questions). For each question the patient can choose between: not at all (0 points), mild (1 point), moderate (2 points) and severe (3 points) [21]. Patients missing 6 or more questions in the IIQ were excluded from further analyses (N = 1).

To evaluate women’s overall appraisal of the severity of their UI we asked the patients to rate their experience of the severity of their incontinence problem with the Patient Global Impression of Severity (PGIS) into one of four categories (negligible, mild, moderate, severe) [20].

### Sample size calculation

Improvement in the severity of the incontinence was estimated to occur in 40 % of those in the treatment group compared to 5 % in the group without intervention. Giving a significance level of 3 % and a power of 80 %, 40 participants were needed. We expected a drop out during the trial of 30 % and therefore we set our target at 60 patients.

### Statistical methods

To test for the changes on the total IIQ-scores between T0 and T1 we used paired t-tests. To assess the effect of the treatment on the Sandvik subgroups- and PGIS-scores we used the McNemar test. [22] In order to test for the possible influence of age, the type of incontinence on the effect measures of the treatment we used a General Linear Model (univariate analyse of variance) with the dependent variables age and type of incontinence and the independent variables total Sandvik-. IIQ-and the PGIS-scores.

To investigate differences between the drop-out (patients who started the treatment but dropped out during the follow up of three months) and the group patients who completed treatment we used a General Linear Model (univariate analyse of variance) with the dependent variables age, the total Sandvik- and IIQ-scores and Chi-square tests for variables type of incontinence, co-morbidity, use of medication and PGIS-scores.

### Ethical approval

Upon consultation, the Medical Ethics Committee (CMO region Nijmegen Arnhem) stated that ethical approval was not necessary because of the non-invasive character of the study (CMO-nr 2010/460).

## Results

### Descriptive data

One hundred and three women entered the treatment program. Thirty-three of them dropped out before the 3 months appointment. Additionally, three patients stopped treatment before the three month appointment because the UI sufficiently improved or they didn’t fill out the T1 questionnaires (Fig. 1). The age of the study populations varied between 24–87 years (mean 55.0, SD 12.6). Fifty two (50.2 %) patients were diagnosed with stress-incontinence, 15 (14.7 %) with urge incontinence and 36 (34.8 %) with mixed incontinence (Table 1). Most patients (78 %) suffered from a mild to moderate severity, in 22 % the UI was (very) severe. Thirty five percent of the patients experienced a negligible impact of the UI on daily life, 59 % a mild to moderate impact and two percent a severe impact. Despite the negligible impact of the UI in a part of the women they chose to undergo a treatment because the kind of treatment was not invasive. Table 1 summarizes the co-morbidities and use of medication. For reasons of comparison Table 1 also shows the prevalence of co-morbidities and use of medication in a Dutch standard population according to the CMR. Co-morbidities and the use of antidepressants were more prevalent in the study population compared to the standard population.

**Table 1:** Patient characteristics N = 103

Characteristic	N (%)	Presented morbidity in women (age 45–65) in standard population (CMR)^a^ (%)
Age mean (SD)	55.0 (±14.6)	
Incontinence		
Stress	52 (50.5)	
Urge	15 (14.7)	
Mixed	36 (34.8)	
Severity of UI (Sandvik score)		
Mild	11 (10,7)	
Moderate	69 (67,0)	
Severe	22 (21,4)	
Very severe	1 (0,9)	
Impact on daily life (IIQ)		
Activities	1.61 (SD 2.50)	
Social	1.32 (SD 2.62)	
Emotional	1.37 (SD 2.07)	
Travelling	1.76 (SD 2.54)	
Impression of severity (PGIS)		
Negligible	36 (34,9)	
Mild	40 (39,2)	
Moderate	23 (19,8)	
Severe	4 (2,1)	
Co-morbidity		
Neurologic disease	8 (7.5)	(1.9)^b^
COPD/chronic cough/asthma	10 (9.4)	(6.8)
Diabetes mellitus	8 (7.5)	(4.3)
Heart failure	4 (3.8)	(2,0)
Hypertension	25 (23.6)	(14.5)
Obesity	27 (25.5)	(11.9)
Use of medication		
Diuretics	15 (14.2)	(14.7)^c^
Antidepressants	12 (11.3)	(2.1)^d^
Other	29 (27.4)	^−^

^a^CMR: Continuous Morbidity Registration Nijmegen

^b^TIA/CVA, Parkinson’s disease, epilepsy, ^c^Hypertension, heart failure, ^d^Depressive disorder

Sixteen percent of the women declared they were not taking their time for micturition and thirty-seven (34.9 %) patients regularly postponed micturition. In addition, seven (6.8 %) patients had mobility constraints. The average number of caffeine intake was 3.6 cups a day (SD 2.6) and the average number of alcohol intake was 0.8 units a day (SD 1.5), which is quite common in the Netherlands.

Not all patients completed all questionnaires. Fifty-seven (85.1 %) patients completed the Sandvik-score at intake and 3 months. Forty-eight (71.6 %) and 46 (68.7 %) patients completed the IIQ and PGIS at both measurements.

### Outcome data

**Fig. 2:**
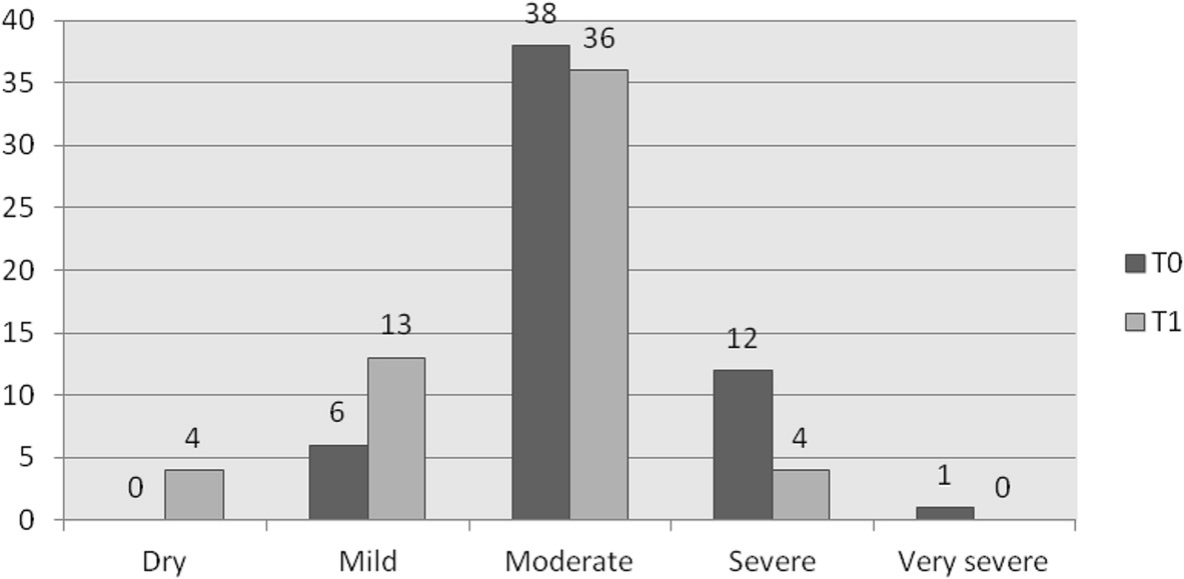
Number of patients by severity categories (Sandvik) at T0(baseline) and T1(3 months) N=57

**Fig. 3:**
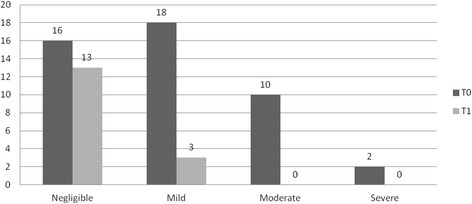
Number of patients by impressed severity categories ( PGIS) at T0 (baseline) and T1 (3 months) N=46

Between baseline and 3 months treatment the Sandvik categories of severity significant improvement *(P = 0.005)*: 23 patients (40.4 %) improved, 3 patients (5.3 %) deteriorated and 31 patients (54.4 %) stayed in the same sub group (Fig. 2). The total IIQ-score improved 2.54 points *(P = 0.012)* (Table 2), whereas the impact on daily activities improved 0.73 points *(P = 0.032)*, on social functioning 0.60 points *(P = 0.030)* and on emotional well-being 0.63 points *(P = 0.031)*. Although the impact on travelling and mobility improved, this difference was not significant *(P = 0.082*). Also the PGIS-score improved significantly (*P = 0.029):* there was an improvement for 19 patients (41.3 %), a deteriorated in 4 patients (8.7 %) and in 23 patients (50.0 %) the PGIS did not change (Fig. 3). Age or type of incontinence did not influence the effect of treatment.

**Table 2:** Impact on quality of life (IIQ-scores) at T0(baseline) and T1(3 months) N = 48

	Intake (T0) (SD)	3 months (T1) (SD)	Change	Paired *T*-test (*p*-value)
IIQ total	6.05 (8.41)	3.51 (6.03)	2.54	0.012*
IIQ activities	1.61 (2.50)	0.88 (1.57)	0.73	0.032*
IIQ Social	1.32 (2.62)	0.72 (1.72)	0.60	0.030*
IIQ emotional	1.37 (2.07)	0.74 (1.56)	0.63	0.031*
IIQ travelling	1.76 (2.54)	1.19 (2.00)	0.57	0.082

* *P* < 0.05

We compared the drop-out group with the group that completed treatment to determine whether there were any differences in patient’s characteristics, severity of involuntary urine loss and impact of UI on the quality of life. No differences were found between the group that competed treatment and the drop-out group according to age, type of incontinence, co-morbidity, use of medication, Sandvik-scores, IIQ-scores and PGIS-scores. The group that competed treatment contained significantly more patients with hypertension *(P = 0.023)*. The total IIQ-score was significantly higher in the drop-out group *(P = 0.036)* compared to the group that completed treatment. The same applied for the IIQ-scores for social functioning *(P = 0.015)* and emotional well-being *(P = 0.015fig*.

Women mentioned several reasons for not completing treatment (Table 3). Ten patients were not satisfied about the improvement of UI; five of them were referred to a physiotherapist and five to an urologist/gynecologist. Five patients experienced difficulties in performing the exercises or in adherence to the treatment program. Five patients stopped the program because of other health problems or others priorities, like being strongly involved in the care of a sick husband or too busy with the revalidation after a total hip replacement. Lastly, 12 patients did not show up for control without a known reason.

**Table 3:** Reasons for non completing treatment N = 33

Reason	N
Lack of improvement, referral to physiotherapist	5
Lack of improvement, referral to urologist/gynaecologist	5
Difficulties performing exercises or adherence to the program	5
Other health problems	4
Other priorities	1
Unrealistic expectations of the program	1
Reasons unknown	12

## Discussion

### Key results

Our first important finding is that treatment by trained nurse practitioners seems to improve the severity of the incontinence and quality of life especially on social functioning and emotional well being after three months of therapy.

Secondary, patients who dropped out from treatment did not differ in age and type of UI compared to the observational group but experience more social and emotional impact of the incontinence. The most important reason for discontinuing treatment is lack of improvement. The in our study group experienced minimal impact of the UI will also be a reason why patients are not always motivated to do exercises for a long time.

Finally, inadequate micturition habits are very common in the observational group Fortunately the nurse practitioner has time to discuss these risk factors and time to give health education and advice specially focused on UI.

### Interpretation of the results

The improvement of UI after a nurse practitioner treatment is according to previous studies but the differences with our study are that these studies are conducted outside primary care practices [14–16, 23, 24]. Our results are also in line with a systematic review stating that nurse practitioners involved in the care of patients in general lead to an improvement of health outcomes and patients satisfaction [24].

Concerning the drop-out one can expect that especially older women or women with more serious incontinence or women who perceive more impact of UI on daily life will not complete treatment. Our study supports this hypothesis only for the impact of UI on quality of life. The total IIQ-score and the IIQ-scores for social functioning and emotional well-being are significantly higher in the drop-out group meaning that they strongly suffer from their incontinence. It takes time to achieve any improvement by treatment and because of the severe impact it may be too difficult or not appropriate to them to wait for improvement.

The lack of improvement as reason for drop-out seems to be quite logical but is scarcely mentioned in the literature [10, 24–28]. Patients also experience difficulties in performing exercises or have to face other health problems. These finding are in line with other studies [10, 25–28].

Concerning the high prevalence of inadequate micturition habits. As already mentioned the nurse practitioner is competent and has the time to discuss these risk factor. Moreover the nurse practitioner is trained to perform motivational interviewing to motivate patients to change life style like micturition habits. The prevalence of mobility constraints and the intake of caffeine and alcohol are not extremely high. It’s important to take attention to these factors because they negatively affect incontinence.

### Limitations

Before drawing any conclusions on the basis of our findings the following needs to be considered.

The dropout rate in this observational study of (32 %) is high but comparable with other studies in which the drop-out rates vary from 11 % to 50 % [14, 15, 23].

A limitation of the current study is that there is no control group involved as in a randomized controlled trial which received usual care from the GP. Several published randomized controlled trial conclude that a control-group without treatment shows no or a slightly improvement of the incontinence [29]. Further research needs to be done to determine whether the nurse practitioner guidance is superior to usual care.

Furthermore as our study focused on the short term effect of the nurse practitioner guidance, more research needs to be done to determine the long term effects.

### Generalisability

A trained nurse practitioner involved in the guidance of women with UI seems to have a positive effect on the severity of the UI and the impact of the UI on the quality of life after three months therapy. A major advantage of the Dutch nurse practitioners is that they are working in the GP’s office. Therefore they are familiar to cooperate with the GP. This facilitates nurse practitioners to consult a GP easier when necessary, probably lowers thresholds for patients to seek for help and possibly increases patient’s motivation. Former research shows that patient’s motivation is one of the most important factors for a successful treatment of UI [9]. Nurse Practitioners can enhance this motivation and thereby increase the effect of treatment. Another advantage of nurse practitioners is that they actively can ask the patient with diabetes or chronic pulmonary disease about UI. Both morbidities are known with a high prevalence of UI and both morbidities are in the Netherlands mostly in care of the nurse practitioner [17].

One of the biggest problems of conservative treatment in case of UI is the high withdrawal of the patients. There are several options to improve the approach of UI by involving a trained nurse practitioner. First of all, exercises should be discussed with patients in a comprehensive way. Nurse practitioners should check several times whether patients still know how to perform the exercises and feel comfortable about it. It is also important that nurse practitioners keep in mind that the training program for patients with UI is time-consuming, not always easy to sustain and difficult to implement in daily life. This applies particularly patients with other health related problems.

Secondly, it is also important to discuss patient’s expectations about the aim of the guidance by the nurse practitioner. This provides an opportunity to prevent patients for having unrealistic expectations, thereby avoiding possible disappointments and referrals to specialists before the treatment is finished.

## Conclusion

In conclusion a trained nurse practitioner involved in the guidance of women with UI seems to have a small positive effect on the severity of the UI and the impact of the UI on the quality of life after three months of therapy, especially in patients with little complains. Patients who do not complete treatment experience more impact of the UI on social function and emotional well being. We have to keep in mind the high rate of drop-out. Most important reasons for drop out are lake of improvement and difficulties in performing exercises. Also it is not clear if the improvement is due the nurse practitioner or the ‘treatment’ itself. More research needs to be done in order to determine the long term effects of the nurse practitioner guidance and to determine whether the guidance is superior to a control group with for example usual care.

## Abbreviations

GP: General Practitioner
IIQ: Incontinence impact questionnaire
PGIS: Patient global impression of severity
UI: Urinary incontinence
CMR: Continues morbidity registration nijmegen
ICI: International consultation on incontinence
